# Parental postpartum affective disorders as a risk factor for infant bedtime resistance

**DOI:** 10.1192/j.eurpsy.2021.1482

**Published:** 2021-08-13

**Authors:** B. Ragni, S. De Stasio, T. Grimaldi Capitello, R. Giampaolo, S. Gentile

**Affiliations:** 1 Human Studies, LUMSA University, Rome, Italy; 2 Clinical Psychology, Bambino Gesù Children Hospital, Rome, Italy; 3 Department Of Pediatrics, Bambino Gesù Children’s Hospital, Rome, Italy

**Keywords:** paternal involvement, Postpartum depression, sleep

## Abstract

**Introduction:**

Infant intrinsic factors, parental mental health, and parenting functioning could influence infant sleep development (Camerota et al., 2019). The current study was designed to advance understanding of parental mental health in influencing bedtime resistance in infants aging 8-12 months.

**Objectives:**

The main aim of the present study was to examine the role of parental postpartum affective disorders, infants’ temperament and paternal involvement at bedtime in predicting infants’ bedtime resistance (e.g. fussing, crying or protesting).

**Methods:**

60 Italian families of infants (34 boys and 26 girls) aging from 8 to 12months (M =10.73, SD = 2.54) participated in this study. Parents completed Brief Infant Sleep Questionnaire (Sadeh et al., 2009), Perinatal Assessment of Paternal and Maternal Affectivity (Baldoni et al., 2018), QUIT for infants’ temperament (Axia, 2002) and an ad-hoc questionnaire for fathers’ involvement. Two multiple linear regressions (MR), one for fathers and one for mothers, and relative weight analyses (RWA) were conducted.

**Results:**

Infants’ involvement in constant bedtime routines (reported by fathers: β = −.35, p < .05; mothers: β = −.31, p < .05) and paternal involvement at bedtime (fathers: β = −.45, p < .01; mothers: β = −.27, p < .05) represented protective factors for infants’ bedtime difficulties. Paternal affective disorders, accounted for 17.2% of the explained variance for mothers’ and 12.5% for fathers’ reports of infant bedtime difficulties, more than did maternal postpartum affective disorders.

**Conclusions:**

Findings support that parental mental health can interfere with infants’ bedtime resistance.

